# 多色标记免疫组织化学染色和免疫荧光染色在肺癌免疫治疗中的研究进展

**DOI:** 10.3779/j.issn.1009-3419.2020.102.47

**Published:** 2021-01-20

**Authors:** 文佳 孙, 建娅 周, 建英 周

**Affiliations:** 310000 杭州，浙江大学医学院附属第一医院呼吸与危重症医学科 Department of Respiratory Disease, Thoracic Disease Center, The First Affiliated Hospital, College of Medicine, Zhejiang University, Hangzhou 310000, China

**Keywords:** 肺肿瘤, 多色标记, 免疫组织化学, 免疫荧光, 免疫治疗, Lung neoplasms, Multiplex, Immunohistochemistry, Immunofluorescence, Immunotherapy

## Abstract

肺癌是目前临床上最常见的恶性肿瘤，严重威胁着患者的生命健康及生活质量。程序性细胞死亡受体1（programmed cell death receptor 1, PD-1）及其配体（programmed cell death ligand 1, PD-L1）抑制剂为非小细胞肺癌（non-small cell lung cancer, NSCLC）患者提供了新的治疗策略。现有的生物标志物检测对准确选择免疫治疗受益的患者均有一定的价值，但都存在着局限性。多标记免疫组织化学/免疫荧光（multiplex immunohistochemistry/immunofluorescence, mIHC/IF）技术允许在单一组织切片上同时检测多个抗体，并对细胞组成、细胞功能和细胞-细胞相互作用进行全面研究。国内外已有大量研究使用mIHC/IF技术对肿瘤免疫微环境（tumor immune microenvironment, TIME）下特异性免疫细胞群进行了探索，发现其有助于肺癌患者临床预后判断及疗效预测。肺癌免疫治疗时代，这项技术在转化研究和临床实践中均具有良好的应用前景。本文就mIHC/IF检测方法在肺癌免疫治疗中的研究进展进行了总结和展望。

## Abstract (en)

Lung cancer is the most common malignancy and is a major public health problem worldwide. Programmed cell death receptor 1 (PD-1)/programmed cell death ligand 1 (PD-L1) inhibitors provide a new treatment strategy for non-small cell lung cancer (NSCLC). The existing biomarkers all have advantages and defects in selecting patients who benefit from immunotherapy. The multiplex immunohistochemistry/immunofluorescence (mIHC/IF) provides multiplex staining, allows comprehensive studies of cellular composition, cellular functions and cell-cell interactions. A number of studies have used mIHC/IF to explore specific immune cells in tumor immune microenvironment (TIME) and found that it is helpful for clinical prognosis and efficacy prediction in patients with lung cancer. In the era of immunotherapy for lung cancer, this technique has a bright future in translational research and clinical practice. The research progress of mIHC/IF detection in lung cancer immunotherapy is summarized in this review.

肺癌是临床常见的恶性肿瘤，其发病率及死亡率均居恶性肿瘤的首位，严重威胁着患者的生命健康及生活质量^[1]^。由于早期诊断率低，大部分患者在诊断时已是晚期。目前局部晚期或发生远处转移的患者5年生存率分别只有26%及4%^[2]^。近年来，免疫检查点抑制剂（immune checkpoint inhibitors, ICIs），特别是程序性细胞死亡受体1（programmed cell death receptor 1, PD-1）及其配体（programmed cell death ligand 1, PD-L1）抑制剂因其普适性、显著的抗肿瘤活性以及良好的安全性，提高了晚期非小细胞肺癌（non-small cell lung cancer, NSCLC）患者的预后，受到了广泛的关注^[3]^。然而PD-1/PD-L1抑制剂的疗效并不是在所有患者中都很理想，而且可能伴发严重的免疫相关不良事件（immune-related adverse events, irAEs），甚至危及生命^[4]^。目前已有的生物标志物对肺癌患者预后及疗效预测均有一定的价值，但都存在着局限性与不足，需要开发更有效的生物标志物，以优化患者利益，并指导治疗。

许多新发现的生物标志物，特别是肿瘤免疫治疗的生物标志物，都与肿瘤免疫微环境（tumor immune microenvironment, TIME）有关^[5]^。TIME是肿瘤和免疫系统之间复杂的动态交叉作用的结果，实体肿瘤的TIME包括瘤内免疫细胞的密度、定位和组成等^[6, 7]^。越来越多的研究^[6]^表明，认识TIME下免疫和肿瘤相关分子的表达模式和功能，对于选择最有可能受益于免疫治疗的患者群体至关重要。传统的免疫组织化学染色/免疫荧光染色（immunohistochemistry/immunofluorescence, IHC/IF）是目前TIME研究最常用的检测方法，在肺癌的病理类型和生物标志物的评估中发挥着至关重要的作用，能够协助临床医生及时、准确地做出治疗决策，但仍存在许多局限性，必须开发更加可靠和高效的免疫组化系统^[8]^。最近一种新的多标记免疫组织化学染色/免疫荧光染色（multiplex immunohistochemistry/immunofluorescence, mIHC/IF）技术能够在一个组织切片上获得多种生物标志物，同时获得关于细胞组成和空间排列的多通道信息，对TIME进行高维分析。近来使用mIHC/IF技术对TIME下特异性免疫细胞群作为肺癌患者治疗预后及疗效预测因素的研究取得了一定的进展，本文对此作一综述。

## 1 mIHC/IF检测

传统的IHC/IF检测是用酶或荧光标记的抗体对福尔马林固定及石蜡包埋（formalin fixation and paraffin embedding, FFPE）的样本进行染色，显示特定目标抗原在组织中的表达和定位分布，是目前被广泛应用于TIME研究的组织病理学诊断技术。

mIHC/IF的染色流程与传统的IHC/IF相近，只是在每一轮染色过程中间增加了一个抗体洗脱步骤，通过顺次单标、多轮复染的方式实现在同一张FFPE切片上同时检测多个生物标志物。一种名为多路免疫组织化学单片连续染色（multiplexed immunohistochemical consecutive staining on single slide, MICSSS）的技术较早地被开发出来并广泛运用，该技术通过重复循环的免疫过氧化物酶标记、图像扫描以及显色底物的洗脱，最后进行图像的对齐整合处理来实现多标记染色（图 1）^[9]^。随后，另一种Opal多标记免疫荧光染色技术也被开发出来，利用酪胺信号放大（tyramine signal amplification, TSA）技术的荧光信号与抗原共价键结合不受微波加热的影响，通过重复循环的TSA荧光染色、微波加热去除抗体但保留荧光信号，从而实现7色-9色标记^[10, 11]^。后期处理将混杂的各色信号进行识别和拆分，以及单色信号间的自由组合，从而进行单个生物标志物或生物标志物间的分析。随后，几种自动分析的算法被开发出来，能够自动将多标记图像中具有特异性结构的目标组织区域识别分割开来，并针对感兴趣区域进行定量统计分析，从而实现定量评估细胞免疫表型和免疫细胞的功能定位，同时提供组织背景和空间分布信息^[12, 13]^。目前，一种“组织成像质谱流式系统”的新技术被开发出来，将质谱流式（mass cytometry, CyTOF）超高检测通道数量的优势运用到组织成像研究领域，用稀有金属同位素标记的抗体染色，在同一张FFPF切片上可以获得高达40种蛋白标志物的图像数据^[14]^。

**图 1:**
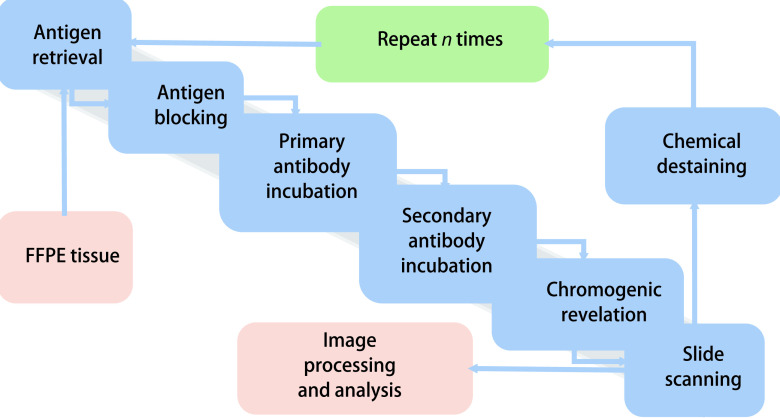
多路免疫组织化学单片连续染色流程图 The multiplexed immunohistochemical consecutive staining on single slide (MICSSS) workflow. FFPE: formalin fixation and paraffin embedding.

## 2 mIHC/IF检测的优势与局限性

### 2.1 传统IHC/IF检测的局限性

传统的IHC/IF检测最大的局限性是在一张FFPF切片上只能染色1个-3个靶标，而精准治疗的肿瘤评估，需要对多个蛋白靶点进行检测，这就需要充足的组织学标本。在大多数病例中，患者的活检样本无法满足除肿瘤病理组织学分型之外的额外检测，这导致错失了从患者样本中获得重要的诊断和预后信息的机会^[15]^。此外，即使有足够的样本进行一系列连续的组织切片传统IHC/IF染色，在多细胞群的研究中对于蛋白之间的相关性也无法准确评估^[16]^。因此，虽然IHC/IF是一种实用且成本效益高的检测方法，但这种方法不能说明复杂TIME的全部情况。

传统的IHC/IF的另一大限制是观察者之间的高变异性，其结果判读主要通过人为定性或半定量，有一定的主观性。例如，Ki-67是多种恶性肿瘤的预后生物标志物^[17]^。但在2017年圣加仑国际专家共识会议上，专家们提出IHC用于Ki-67检测的可重复性的问题及其对临床决策的影响。为减少主观性的影响，目前国际上存在共识，要求实验室具备经验丰富的病理学专家。此外，有研究^[18]^表明，使用可重复且定量的数字分析对Ki-67进行评分，可以消除观察者间的差异。

### 2.2 mIHC/IF检测的优势

mIHC/IF检测实现了在FFPE组织切片上进行多个生物标志物检测，配合定量分析软件，能够自动区分肿瘤和非肿瘤组织，客观地分析多个生物标志物以及细胞组成、功能状态和细胞-细胞相互作用，具有高重复性、高效率和高成本效益的优点^[12, 13]^。

最近，国际上的多项研究探究了mIHC/IF检测较传统方法的准确性和可行性。新加坡学者^[19]^收集了25例三阴性乳腺癌、12例NSCLC和8例其他实体肿瘤的组织，分别用传统的IHC检测方法标记3种PD-L1抗体（22C3、SP142和SP263）的表达情况，然后由4位病理学专家评估染色百分比；mIHC/IF检测方法同时标记3种PD-L1抗体（22C3、SP142和SP263）的表达情况，使用光谱成像设备采集图像并通过软件进行定量分析。该研究评估两种检测方法结果之间的一致性，包括联合阳性分数（combined positive score, CPS）、肿瘤细胞阳性比例分数（tumor proportion score, TPS）及免疫计数（immune count, IC）评分，结果显示，两种检测方法之间存在中-强相关性，一致性达到67%-100%，斯皮尔曼秩相关系数高达0.88。该研究证明了mIHC/IF检测与传统的IHC检测相比，PD-L1定量检测的一致性良好，并且前者更具客观性。美国学者^[13]^对最常见的预测PD-1/PD-L1抑制剂疗效的生物标志物PD-L1 IHC、肿瘤突变负荷（tumor mutation burden, TMB）、基因表达谱（gene expression profiling, GEP）和mIHC/IF检测进行了预测准确性分析。研究纳入了24篇研究，共10余种实体肿瘤8, 135例患者，使用受试者工作特征曲线（receiver operating characteristic curve, ROC）进行预测准确性的评估发现，mIHC/IF检测的曲线下面积（area under the curve, AUC）（0.79）较PD-L1 IHC（AUC为0.65，*P* < 0.001）、GEP（AUC为0.65，*P*=0.003）及TMB（AUC为0.69，*P*=0.049）更高，即mIHC/IF检测的预测准确性（加权/非加权AUC为0.790/0.872；95%CI：0.650-0.688/0.657-0.710）高于其他的检测方法^[13]^。此外，他们发现mIHC/IF检测的阳性预测值（positive predictive value, PPV）和阳性似然比（positive likelihood ratio, LR+）显著高于其他生物标志物，即mIHC/IF出现假阳性结果可能性更低^[13]^。该研究阐明了mIHC/IF检测在预测患者免疫治疗的疗效方面的准确性高于目前的PD-L1 IHC、TMB及GEP检测方法，意味着其在未来的临床应用上将有较大潜力。

### 2.3 mIHC/IF检测的局限性

在技术上，mIHC/IF仍然是一个基于IHC的平台，也面临着抗体交叉反应以及光学交叉问题。为了克服抗体交叉反应，mIHC/IF检测采用抗体剥离的方法，早期的MICSSS技术经过化学方法洗脱抗体，容易造成组织样本损伤，特别是抗原丢失。较新的Opal多标记免疫荧光染色技术通过微波加热去除抗体，此过程与抗原修复过程相似，减小了对组织样本及抗原的损伤。此外，该技术相比MICSSS技术，不需要图像后期对齐整合，多种荧光信号同时存在于一张图像上，改善了标记物间定位的准确性和稳定性，但面临着光谱串扰的问题。对于三色以下的复染图像，可以通过普通显微镜采集，但对于更多色的复染图像，由于光谱串扰，需要采用更加专业的光谱成像设备以及定量分析软件进行图像采集和分析，昂贵的费用限制了这种方法的广泛使用^[20]^。而mIHC/IF检测平台作为一个临床转化平台，其分析速度、周转时间、计算能力、服务器和存储也对临床病理实验室具有较高的要求。

此外，传统的IHC/IF检测是在整个组织中进行评分，而mIHC/IF检测捕捉整个组织切片中感兴趣的区域进行分析，很可能因为组织异质性而导致结果与实际情况存在偏差，存在组织学定量不准确或错过罕见事件的可能。目前，mIHC/IF检测对于整个组织切片的分析正在测试中^[19]^。

## 3 mIHC/IF技术在肺癌免疫治疗中的临床价值

近年来，肺癌治疗已经进入精准医疗的时代，其中，有效的生物标志物检测是准确选择获益人群的关键环节。Remark等^[9]^和Tsujikawa等^[21]^研究发现，在癌症患者中，ICIs治疗前后TIME的高维特征与治疗反应相关。国内研究^[6]^发现，TIME内的肿瘤浸润淋巴细胞（tumor infiltrating lymphocytes, TILs）与肿瘤细胞的相互作用密切，能够促进或阻止肿瘤的生长和侵袭，在肺癌中具有预后及疗效预测价值。由于TILs是一群具有较大异质性的细胞群体，因此需要进一步细化及量化具体指标。mIHC/IF检测能够同时分析多个生物标志物，客观地评估TILs亚群。

### 3.1 TILs亚群的密度

目前的临床实践中，肺癌患者的结局主要依据肿瘤原发灶-淋巴结-转移（tumor-node-metastasis, TNM）分期、病理组织学分型和相关生物标志物等。近几年，国际上采用mIHC/IF检测探究不同TILs亚群的密度用于判断肺癌临床结局已有不少报道。目前，已有多项研究使用mIHC/IF检测采用不同生物标志物组合对NSCLC患者的TILs亚群进行探索，发现高密度的CD3^+^CD8^-^ TILs亚群和CD8^+^ TILs亚群，与NSCLC患者较长的总生存期（overall survival, OS）相关，其中，CD8^+^ TILs是独立于年龄、肿瘤大小、组织学和肿瘤分期的预后因素^[22-24]^。2018年，意大利学者^[25]^收集了100例早期NSCLC患者，包括42例腺癌（adenocarcinoma, ADC）和58例鳞癌（squamous cell carcinoma, SCC）的手术切除标本和26例接受那武利尤单抗（Nivolumab）免疫治疗的晚期NSCLC患者（13例ADC、13例SCC）的组织标本，通过mIHC/IF检测发现PD-1低表达的CD8^+^ TILs亚群的高密度与手术切除患者较长的OS相关（HR=2.268, 95%CI: 1.056-4.871, *P*=0.03），且与Nivolumab治疗的晚期NSCLC患者较长的无进展生存期（progression-free survival, PFS）相关（HR=4.51, 95%CI: 1.45-13.94, *P*=0.004）。并且，100% Nivolumab治疗有临床获益的患者（*n*=9）均显示较低的PD-1与CD8的比值，而仅有21.4%疗效差的患者（*n*=17）显示低比值（*P* < 0.001）。提示PD-1低表达的CD8^+^ TILs亚群对手术切除患者的预后以及晚期NSCLC患者对免疫治疗的反应均有积极影响。同年，美国学者^[26]^对39例接受不同PD-1/PD-L1抑制剂单药免疫治疗的NSCLC患者及110例未接受免疫治疗的NSCLC患者进行mIHC/IF检测评估发现，接受免疫治疗的NSCLC患者中，19例获得持久临床获益患者的CD3^+^ TILs密度较23例疗效差的患者高出2.4倍（*P*=0.02），并且，“休眠”的CD3^+^ TILs细胞亚群（即低活化低增殖的CD3^+^ TILs）密度越高，NSCLC患者免疫治疗的PFS（*P*=0.043）及OS（*P*=0.003）越长；而在未接受免疫治疗的NSCLC患者中，“休眠”的CD3^+^ TILs细胞亚群与较长的生存获益没有相关性，这一发现证实了该细胞亚群可能是免疫治疗反应的预测生物标志物，但不是预后标志物。

美国学者Carvajal-Hausdorf等^[27]^利用mIHC/IF检测对90例小细胞肺癌（small cell lung cancer, SCLC）患者的TILs探索发现，与NSCLC患者预后显著相关的CD8^+^ TILs亚群，与SCLC患者的预后无显著相关性（*P*=0.16）。相似地，挪威学者Rakaee等^[28]^通过mIHC/IF检测对536例NSCLC患者研究表明，瘤内高密度的CD66b^+^肿瘤相关中性粒细胞（tumor-associated neutrophils, TANs）在SCC患者中是一个预后良好的独立预测因素（*P*=0.038），而在ADC患者中则是预后不良的独立预测因素（*P*=0.032）。这些研究提示了同一种TILs亚群在不同病理组织学类型的肺癌患者中对肿瘤-宿主免疫活动有不同的影响，这可能与基因组及基质异质性有关，许多人认为不同组织类型肺癌是不同的实体。法国学者对于驱动基因阳性的肺癌亚群患者进行深入研究发现，ALK阳性的NSCLC患者PD-L1表达及瘤内CD8^+^ TILs密度均高于EGFR阳性患者（*P*=0.03）和野生型患者（*P*=0.012），这个结果表明EGFR突变患者不易对免疫治疗产生反应，而ALK可能是抗PD-1/PD-L1抑制剂治疗的潜在靶点^[29]^，但临床研究^[30]^未能证实ALK阳性增加NSCLC患者对PD-1/PD-L1抑制剂治疗的敏感性。这表明在该群体中可能还存在其他的耐药机制，如T细胞的抑制受体的共同表达或免疫抑制细胞的浸润^[10, 11]^。

此外，mIHC/IF检测的多参数分析可以更好地显示传统方法无法检测到的复杂细胞表型的TILs亚群。最近发现的一种存在于组织中不参与循环的记忆CD8^+^ T细胞亚群，被称为组织常驻记忆T（tissue-resident memory T, T_RM_）细胞，是一种包括多个生物标记（如CD103、CD49a、CD69）的复合表型细胞^[31]^。mIHC/IF检测发现，T_RM_细胞能够在上皮肿瘤区域内与肿瘤细胞密切接触，在肺癌等多种人类恶性肿瘤中的浸润与较好的临床预后相关，且在免疫监测中发挥了重要作用^[31, 32]^。T_RM_细胞在不同的临床研究中被证实是比CD8^+^ TILs更好的预后标记^[33, 34]^。

### 3.2 TILs亚群的定位和空间关系

目前，较多mIHC/IF检测相关研究发现TILs亚群的定位及空间关系也有较大的临床价值。Parra等^[35]^通过mIHC/IF检测112例NSCLC患者（接受新辅助化疗患者61例，未接受新辅助化疗患者51例）的组织样本发现，接受新辅助化疗的NSCLC患者中，CD4^+^CD3^+^ TILs的定位与患者的生存相关，其在上皮内的高密度与患者更长的OS获益相关（*P*=0.048），而在基质内的高密度与患者的OS无明显相关性。TILs亚群与肿瘤细胞、不同TILs亚群间的空间关系能更全面地评估患者预后，例如，Barua等^[36]^通过mIHC/IF检测120例NSCLC患者的组织样本发现，肿瘤核心区域Foxp3^+^ TILs（调节性T细胞）增加是NSCLC患者预后总生存较差的独立因素（HR=1.52, 95%CI: 1.11-2.07, *P*=0.009），但Foxp3^+^ TILs间的CD8^+^ TILs缓解了这一效应，反而与患者较好的OS显著相关（HR=0.96, 95%CI: 0.92-0.99, *P*=0.042）。此外，Mezheyeuski等^[37]^还发现CD8^+^ TILs与肿瘤细胞的距离、CD8^+^ TILs/Treg细胞比值与NSCLC患者预后密切相关。由此，我们推定免疫细胞的定位及其空间关系，都与肺癌预后有着密切的关系，这可能与空间接近的细胞间的相互作用相关。

### 3.3 TILs亚群上的分子表达

mIHC/IF检测相关研究发现除TILs亚群的密度、定位及空间关系外，TILs亚群上相关分子的表达与肺癌患者的临床结局也有较大的关系。美国学者Zugazagoitia等^[38]^利用mIHC/IF检测进一步分析了69例接受免疫治疗的NSCLC患者和258例未接受免疫治疗的NSCLC患者，发现接受免疫治疗的NSCLC患者较长的OS与CMTM6和PD-L1在CD68^+^ TILs亚群（巨噬细胞）中的高共表达显著相关（HR=0.38, 95%CI: 0.16-0.92, *P*=0.03），但与其在肿瘤细胞中的高共表达无明显相关性（*P*=0.15），而此现象在未接受免疫治疗的NSCLC患者中未观察到。类似地，美国学者Liu等^[39]^利用mIHC/IF检测，在425例未接受免疫治疗和62例接受帕博利珠单抗（Pembrolizumab）/Nivolumab/阿特珠单抗（Atezolizumab）治疗的NSCLC患者的检测中发现，单药免疫治疗的NSCLC患者更长的OS与CD68^+^ TILs亚群（巨噬细胞）中PD-L1高表达有关（细胞计数*P*=0.036；分子共定位*P*=0.019），但是与肿瘤细胞（细胞计数*P*=0.29）或者间质细胞（细胞计数*P*=0.71）中PD-L1的高表达无关；而在未接受免疫治疗的NSCLC患者中，CD68^+^ TILs亚群中PD-L1高表达与患者OS无关（*P*=0.16）。PD-L1是最常用于免疫治疗选择的可靠预测性生物标志物，这些研究结果表明，其在巨噬细胞中的表达较肿瘤细胞及间质细胞中的表达有更大的临床价值。由此我们推测，如PD-L1这些常用的生物标志物与TILs的分析相结合，对NSCLC患者免疫治疗疗效的预测可能更具有准确性。

TILs在肿瘤进展中起着关键作用，并依赖于各种TILs亚群的密度、定位和空间关系等，严重影响患者的临床结果。mIHC/IF检测让我们不仅能够分析一种特定的细胞类型，还能整合肿瘤不同部位的免疫细胞之间的关系，通过mIHC/IF检测对NSCLC的TILs亚群深入分析，可以获得更多的预后和预测数据，有助于准确地选择能够从免疫治疗中获益的肺癌患者。尤其在组织学标本稀少的情况下，mIHC/IF检测可以作为新的检测方法选择，有望成为临床上有力的工具。

## 4 总结

综上所述，在肺癌免疫治疗的时代，判断肺癌患者的临床预后以及预测其对免疫治疗的疗效的方法不断创新。传统的IHC/IF检测仍是当今的主流，用以筛选获益人群，但是受到诸多因素的影响，包括患者组织样本有限，染色过程冗长费力，染色结果受病理医生主观影响，数据采集及分析系统落后等。新兴的mIHC/IF检测能够在一个FFPE切片上获得多种生物标志物，同时获得关于细胞组成和空间排列的全面信息。目前的研究显示其在筛选免疫治疗的受益人群上较传统方法有较高的准确性，让我们相信，mIHC/IF检测在未来有着广阔的应用前景^[40]^。当然，在作为临床常规检测前，mIHC/IF检测还需要进行更多抗体的探索，并进行严格的大规模验证研究。
